# Effect of exogenus protease on performance, nutrient digestibility, intestinal histomorphometric, meat quality characteristics, carcass yield in broilers fed low protein diets

**DOI:** 10.1007/s11250-023-03562-y

**Published:** 2023-05-04

**Authors:** C. F. Duque-Ramírez, J. A. Javierre, L. M. Peñuela-Sierra, M. Diaz-Vargas

**Affiliations:** 1 Facultad de Ciencias Agropecuarias, Semillero de investigación SINA, Universidad del Tolima, 730006299, Ibagué, Colombia; 2 Científico de Tekzol SAS, Bogotá D.C., Colombia; 3 Facultad de Ciencias Agropecuarias, Universidad de Ciencias Aplicadas y Ambientales (U.D.C.A), 111166 Bogotá D.C., Colombia

**Keywords:** Protein, Metabolizable energy, Villous, Growth, Crypt

## Abstract

The objective of the present study was to evaluate the effects of increasing doses of protease on broilers from 1 to 42 days of age. A total of 1290 Ross AP broilers were used, distributed among five treatments: positive control diet, negative control diet (NC), NC + 50 ppm of protease, NC + 100 ppm of protease, and NC + 200 ppm of protease. Each treatment contained six replicates of 43 animals each. The inclusion of proteases in the diet had effects (*P *< 0.05) on body weight, feed intake, weight gain, and feed conversion in the 12 to 21 day period; body weight, weight gain, and feed intake in the 29 to 42 day period; nutrient digestibility (energy metabolizability coefficient and crude protein at 28 days); and intestinal parameters (crypt and muscle width of jejunum and ileum at 28 days and villus length, crypt length, and jejunum thickness muscle layer at 42 days). These results indicate that the inclusion of protease in broiler feed can improve production parameters when the amount of crude protein in the diet is reduced.

## Introduction

Poultry is the livestock that provides most of the animal protein for consumption, according to the data given by FENAVI (National Federation of Poultry Farmers of Colombia), in Colombia approximately 1,620,000 tons of broiler consumption are produced per year, which reflects an increase in production and distribution. The growth figure of the poultry sector in 2019 was 4.0%; since the total demand for chicken reached 842 million units, with a monthly average of 70.1 million.

The percentage of digestible protein in broilers is approximately 80% to 85%, lower than that of starch, which is 90% (Zanella et al., 1999), indicating that not all of the protein in the diet is utilized in the gastrointestinal tract, and a small fraction of this is excreted in the feces following the digestive process (Glitso et al., 2012). The undigested protein in the intestine can be transformed by the intestinal microbiota into harmful compounds, such as ammonium, or in the soil into nitrite and nitrous oxide (Weir et al., 2017), which has economic and environmental impact. Therefore, crude protein has been the subject of multiple studies seeking to minimize its inclusion in poultry feed (Borda-Molina et al., 2019) by improving digestibility. Therefore, there is great market interest in taking advantage of undigested protein through the addition of exogenous enzymes such as proteases (Grimes, 2011), favoring the formulation of balanced feed with lower protein levels and thus reducing the cost of the diet (Ding et al., (2016). We aimed of the present study was to investigate the effects of protease at different levels (50, 100 and 200 ppm) on a protein and energy deficient diet and whether this would enhance broiler performance, nutrient digestibility, intestinal histomorphometric parameters, color parameters of meat, carcass yield and cut yield strength in poultry.

## Materials and methods

### Birds management and housing

All experimental procedures in this research were conducted under the guidelines of the Animal Care and Use Committee of Department of Animal Science of University of Applied and Environmental Sciences U.D.C.A., Bogotá D.C., Colombia. The experiments were carried out in the Poultry Farming Sector at the Experimental Farm of Tolima University. A total of 1290, 1-day-old birds Ross AP male broiler divided into five groups with six replicates, were raised in a semi-heated shed, in floor pens of 3.58 m2 (12 birds/m2) with rice husk as litter and equipped with one feeder and one drinker each. The temperature was maintained at 32ºC at placement and was gradually reduced to ensure comfort by using heater, fans, and nebulizers. The lighting program throughout the study consisted of 23 h of light and 1 h of darkness. Feed was provided in mash form, and birds had free access to feed and water libitum throughout the whole experimental period.

### Treatments and diets

The diets were: a control diet (CD) diet meeting all nutritional requirements (Table 1), a negative control diet (NC; diet with -1.0% PB and AA and -50 kcal/kg ME at each stage), NC diet + 50 ppm protease, NC diet + 100 ppm protease, and NC diet + 200 ppm protease. The protease, produced by Aspergillus Niger, was concentrated, granulated, and thermostable. 4-phase feeding was provided, according to the commercial recommendation that is handled in the country. All chicks were fed ad libitum with chick starter crumbs for the first 11 days(pre-starter), and chick feed between 12 and 21 days (starter) and chicken feed between 22 and 28 days (broiler I) and finisher diet between 29 and 42 days (broiler II or finisher). An acid-resistant ash source (Celite) was added at 1% to all diets.

**Table 1:** Ingredients and Nutritional composition of the basal diets

	Start	Grower
	(Day 1—21)	(Day 22—42)
Ingredients (kg)		
Yellow corn	55,01	59,81
Soybean meal 48%	29,86	24,72
Corn gluten meal 60%	6	5
Palm oil	4,07	6,08
DCP	2,19	1,76
Limestone	0,825	0,91
Vit-Min Premix	0,6^a^	0,6^b^
DL methionine 99%	0,34	0,25
Salt	0,24	0,24
Lysine HCl 98%	0,4	0,28
Sodium bicarbonate	0,2	0,15
Choline chloride60%	0,1	0,1
L-threonine	0,12	0,07
Nutrients		
EM Poultry (kcal/kg)	3050	3200
Crude protein (%)	22,25	19,5
Ether extract (%)	6,216	8,17
Linoleic acid (%)	1,475	1,7
Crude fiber (%)	2,305	2,22
Starch (%)	36,995	39,25
Ash (%)	6,94	6,28
Calcium (%)	0,875	0,8
Available P (%)	0,46	0,39
Chloride (%)	0,26	0,25
Sodium (%)	0,195	0,17
Potassium (%)	0,85	0,76
Acid balance (meq/Kg)	228,5	199
Dig Lysine (%)	1,215	1,03
Dig Methionine (%)	0,66	0,54
Dig Cystine (%)	0,3	0,26
Dig Met + Cys (%)	0,95	0,8
Dig Threonine (%)	0,785	0,7
Dig Tryptophan (%)	0,225	0,21
Dig Arginine (%)	1,235	1,08
Dig Valine (%)	0,945	0,86
Dig Isoleucine (%)	0,845	0,72
Dig Leucine (%)	1,705	1,63
Choline (mg/Kg)	1685	1581

^a^Vitamin supplement (content per kilogram of premix): vitamin A 2 916 670 IU, vitamin D3 583 330 IU, vitamin E 8750 IU, vitamin K3 433.33 mg, vitamin B1 408.33 mg, vitamin B2 1333.33 mg, vitamin B12 4166.67 mcg, niacin 8983.33 mg, calcium pantothenate 3166.67 mg, folic acid 200 mg, and biotin 25 mg. Mineral supplement (content per kilogram of premix): iron 12.6 g, copper 3072 mg, iodine 248 mg, zinc 12.6 g, manganese 15 g, selenium 61.20 mg, and cobalt 50.40 mg

^**b**^Vitamin supplement (content per kilogram of premix): vitamin A 2 250 000 IU, vitamin D3 450 000 IU, vitamin E 7000 IU, vitamin K3 418 mg, vitamin B1 300 mg, vitamin B2 1000 mg, vitamin B12 3000 mcg, niacin 7000 mg, calcium pantothenate 2500 mg, folic acid 140 mg, and biotin 14 mg. Mineral supplement (content per kilogram of premix): iron 12.5 g, copper 3000 mg, iodine 250 mg, zinc 12.5 g, manganese 15 g, selenium 75 mg, and cobalt 50 mg

### Performance

Broilers and feed were weighed at 1, 11, 21, 28, and 42 days of age to evaluate performance, which was measured as the feed intake, weight gain, and the feed conversion ratio Mortality was recorded. At 28 and 42 d, twelve birds were selected per treatment (mean ± 5%) stunned by electroshock, and euthanized by decapitation.

### Intestinal morphometry and digestibility

At 28 and 42 d, an approximately 2 cm length of each segment of the small intestine (duodenum, jejunum, and ileum) was collected from two birds per replicate. The samples were placed in flasks containing 10% buffered formalin for tissue fixation. The sections were dehydrated in a series of increasing concentrations of alcohols, freeze dried in xylol, and embedded in paraffin. Semi seriate and cross-sectional 5-mm-thick histological sections were made until five sections per slide were obtained and then stained using the hematoxylin–eosin method. IMAGE PROPLUS 4 computer imaging software was used for morphometric analysis. Twenty-five measurements of villus height, crypt depth and width, and thickness of the muscle layers per unit were carried out, and the villus-crypt ratio (V:C) was calculated (Uni et al., 1998).

The digesta was collected from the lower half of the ileum, frozen, and subsequently dehydrated in a ventilation oven at 55 °C for 72 h. The dried digesta samples and diets were ground in a laboratory mill and sent for analysis of dry matter (DM), gross energy, and nitrogen (N). The DM content was determined at 105 °C for 24 h (AOAC 2006). The total N concentration was determined using the Kjeldahl procedure (No. 984.13) according to AOAC (2007), and the crude protein (CP) was obtained by multiplying the total N by 6.25. The energy values were determined using a Parr 6100 oxygen bomb calorimeter (Parr instrument Co., Moline, IL, USA). For determination of the ileal digestibility coefficients, the indigestibility factor was initially determined using the following formula: Indigestibility factor = % of the indicator in the evaluated diet/% of the indicator in the ileal content. Subsequently, the apparent digestibility coefficient of each nutrient was determined using the following formula: % nutrient in the diet – (% nutrient in the ileal content * indigestibility factor)/% nutrient in the diet * 100.

### Carcass yield

At 43 d of age, two birds were selected per replicate (mean ± 5%) for analysis of carcass yield, choice cuts, and abdominal fat percentage. After an 8 h fast, the birds were stunned by electroshock, euthanized by decapitation, plucked, and eviscerated, and the carcasses were weighed on a digital scale balance to calculate carcass and choice cut yields. Abdominal fat was defined as the fatty tissue surrounding the cloaca, cloacal bursa, gizzard, proventriculus, and adjacent abdominal muscles, as described by Smith (1993); it was then weighed, and its relative weight was calculated in relation to the dressed carcass weight.

### Meat quality characteristics

For the evaluation of meat quality, at 43 d samples of the breast (pectoralis major) and thigh muscles of 12 birds were collected per treatment to evaluate pH, color [L* (lightness), a* (redness), and b* (yellowness)], and cooking weight loss (CWL). Twenty-four hours after slaughtering, the pH of samples was measured using a Testo 205 pH meter (Testo Inc., Sparta, NJ, USA) with a penetration electrode introduced directly into the samples as described by Boulianne and King (1995) and adapted by Olivo et al. (2001). Color was measured using a Minolta CR-400 colorimeter (Konica Minolta Sensing Inc., Osaka, Japan) in three different locations of the breast and thigh according to the methodology described by Van-Laack et al. (2000). The components L*, a*, and b* were expressed using the CIELAB color system. The muscle of the left breast was used for analysis of the CWL.

### Statistical analysis

The data for each parameter were analyzed by the analisis of variance (ANOVA)and polynomial regression, using SAS software (2007). For comparison of the results, statistically signicant diferences among treatments were evaluated using the Tukey at 5% probability.

## Results

In the period from 1 to 11 days, there were no differences in production parameters (Table 2) (*P* > 0.05), while in the period from 12 to 21 days of age, an increasing linear effect (*P* < 0.05) of protease on feed consumption was observed (y = 1251.3 + 0.3236x; R^2^ = 0.8551). A quadratic effect of protease levels was observed (*P* < 0.05) on feed conversion (Y = 1.5981—0.0012x + 0.0000006 × 2; R^2^ = 0.9995), with the lowest value presented at the estimated level of 85.71 ppm, indicating that the best conversion occurs when the inclusion of protease in the diet is 85.71 ppm. In a comparison of the treatments, it was observed that the birds receiving the experimental diets (50, 100, and 200 ppm protease) achieved a feed consumption similar to that of the birds fed the positive control diets. However, the birds that consumed the diet with 100 ppm of protease had a better (*P* < 0.05) FCR during the 12 to 21 experiment days.

**Table 2:** Performance (± standard error) of broilers fed diets containing different protease levels

	Positive control	Negative control	NC + 50 ppm	NC + 100 ppm	NC + 200 ppm	CV (%)	REG
1 to 11 d							
Weight gain (g)	187.28 ± 12.4	197.09 ± 5.4	208.19 ± 5.8	191.42 ± 5.9	198.30 ± 14.4	13.08	Ns
Feed intake (g)	177.84 ± 4.9	182.56 ± 7.2	197.17 ± 4.3	189.32 ± 2.6	193.64 ± 5.9	7.47	Ns
Feed conversion	0.975 ± 0.1	0.924 ± 0.1	0.950 ± 0.1	0.995 ± 0.1	1.010 ± 0.1	15.01	Ns
12 a 21 d							
Weight gain (g)	797.50 ± 7.7	790.19 ± 10.1	813.50 ± 15.4	821.99 ± 7.5	811.76 ± 6.2	3.3	Ns
Feed intake (g)	1327.70 ± 15.1^a^	1261.64 ± 14.5^b^	1263.16 ± 14.1^ab^	1269.66 ± 8.1^ab^	1324.18 ± 14.3^ab^	3	0.02^a^
Feed conversion	1.665 ± 0.1^a^	1.598 ± 0.1^ab^	1.556 ± 0.1^ab^	1.545 ± 0.1^b^	1.632 ± 0.1^ab^	4.19	0.03^b^
22 to 28 d							
Weight gain (g)	426.72 ± 28.1	443.45 ± 34.9	408.11 ± 33.6	395.33 ± 22.1	436.98 ± 20.2	18.02	Ns
Feed intake (g)	709.62 ± 7.8	710.30 ± 11.8	727.97 ± 11.6	700.78 ± 13.8	723.36 ± 19.7	5.09	Ns
Feed conversion	1.694 ± 0.1	1.650 ± 0.1	1.862 ± 0.2	1.796 ± 0.1	1.668 ± 0.1	15.75	Ns
29 to 42 d							
Weight gain (g)	1282.60 ± 19.2	1177.10 ± 18.9	1268.58 ± 22.8	1253.95 ± 7.8	1400.60 ± 27.2	11.09	0.01^c^
Feed intake (g)	2876.60 ± 20.2	2619.90 ± 13.4	2622.55 ± 13.4	2724.59 ± 11.3	2843.65 ± 20.5	5.98	0.01^d^
Feed conversion	2.259 ± 0.1	2.246 ± 0.1	2.091 ± 0.1	2.172 ± 0.1	2.071 ± 0.1	11.89	Ns

Means followed by different letters in the same line differ significantly by Tukey's test (*P* < 0.05). CV = coefficient of variation. REG = regression. Ns = not significant

^a^y = 1251.3 + 0.3236x; R^2^ = 0.8551; ^b^ y = 1.5981 – 0.0012x + 0.0000006x^2^; R^2^ = 0.9995) optimal value = 85.71 ppm; ^c^y = 1184.4 + 1.0365x; R^2^ = 0.9094; ^d^ y = 2544.2 + 1.5589x; R^2^ = 0.9832

In the period from 22 to 28 days of age, no differences were observed (*P* > 0.05), while in the period from 29 to 42 days of age, an increasing linear behavior (*P* < 0.05) was observed in weight gain (y = 1184.4 + 1.0365x; R^2^ = 0.9094) and feed consumption (y = 2544.2 + 1.5589x; R^2^ = 0.9832). In addition, in this period, the birds that received the diet with 200 ppm protease had a higher average weight (5.1% more) than those consuming the positive control diet.

At 28 days, the coefficient of energy metabolizability (Table 3) showed a linear effect (*P* < 0.05) (y = 76.903 + 0.0052x; R^2^ = 0.9956) as dietary protease levels increased. Digestible crude protein had a quadratic response (*P* < 0.05) (y = 17.66 + 0.0503x—0.0002x^2^; R^2^ = 0.622), with the highest value set at the estimated level of 125.75 ppm. A quadratic effect (*P* < 0.05) of protease levels on the metabolizable energy of 42-day-old broilers (y = 3034.6 -3.6324x + 0.012x^2^; R^2^ = 0.9997) was also observed, with the lower value set at the 151.35 ppm level.

**Table 3:** Digestible nutrients, apparent digestibility coefficients and coefficients of energy metabolizability in broilers

	Positive control	Negative control	NC + 50 ppm	NC + 100 ppm	NC + 200 ppm	CV (%)	REG
28 d							
DMD	69.04 ± 0.18ª	68.33 ± 0.045^ab^	66.72 ± 0.045^c^	67.93 ± 0.01^b^	68.74 ± 0.039^ab^	0.26	Ns
CDDM	79.24 ± 0.21ª	78.87 ± 0.052ª	77.80 ± 0.052^b^	78.59 ± 0.012^ab^	78.65 ± 0.044^ab^	0.26	Ns
AME	2912.81 ± 0.28ª	2833.89 ± 0.28^d^	2794.48 ± 0.41^e^	2882.16 ± 0.6^c^	2887.01 ± 0.02^b^	0.026	Ns
CMAME	79.02 ± 0.007ª	76.88 ± 0.22^c^	77.16 ± 0.07^bc^	77.47 ± 0.01^bc^	77.93 ± 0.07^b^	0.29	0.0019^a^
CPD	17.22 ± 0.19^b^	17.13 ± 0.18^b^	21.09 ± 0.24ª	19.74 ± 0.52ª	20.32 ± 0.43ª	4.16	0.023^b^
CDCP	81.15 ± 0.89	78.42 ± 0.86	84.61 ± 0.98	78.30 ± 2.09	82.95 ± 1.75	4.02	Ns
42 d							
DMD	67.48 ± 0.06^b^	67.87 ± 0.001^ab^	68.94 ± 0.21ª	67.14 ± 0.17^b^	67.73 ± 0.09b	0.4	Ns
CDDM	78.55 ± 0.07	78.28 ± 0.002	79.10 ± 0.24	78.57 ± 0.2	78.81 ± 0.11	0.4	Ns
AME	3136.25 ± 0.21ª	3035.60 ± 0.14^b^	2880.13 ± 0.07^c^	2792.97 ± 0.014^d^	2786.1 ± 0.12e	0.009	Ns
CMAME	79.06 ± 0.02ª	77.62 ± 0.14^b^	76.53 ± 0.14^c^	77.02 ± 0.14^bc^	76.79 ± 0.04c	0.29	Ns
CPD	16.47 ± 0.81ª	13.37 ± 0.72^b^	11.99 ± 0.39^b^	14.48 ± 0.3^ab^	12.77 ± 0.19ab	9.03	Ns
CDCP	78.79 ± 2.11ª	73.02 ± 2.35^ab^	70.28 ± 0.97^b^	75.59 ± 0.96^ab^	70.74 ± 0.55ab	4.89	Ns

DMD = digestible dry matter. CDDM = coefficient of digestibility of dry matter. EMA = metabolizable energy. CMEMA = metabolizability coefficient of metabolizable energy. CPD = digestible crude protein. CDCP = crude protein digestibility coefficient. Means followed by different letters in the same line differ significantly by Tukey's test (*P* < 0.05). CV = coefficient of variation. REG = regression. Ns = not significant

Broilers fed the control diet with a higher protein content had higher values for digestible dry matter, dry matter digestibility coefficient, apparent metabolizable energy and the energy metabolizability coefficient at 28 days and apparent metabolizable energy, energy metabolizability coefficient, crude digestible protein and crude protein digestibility coefficient at 42 days than those fed experimental diets. The CN + 50 ppm protease diet had the absolute lowest values for digestible dry matter, dry matter digestibility coefficient and apparent metabolizable energy at 28 days.

At 28 days of age, dietary protease had no effect (*P* < 0.05) on villus height, crypt depth, and the V/C ratio (Table 4) in the jejunum and ileum. However, jejunal crypt width displayed a quadratic response (*P* < 0.05) (y = 69.258 -0.1303x + 0.0005x^2^; R^2^ = 0.957), with the lowest value at 130.3 ppm; likewise, jejunal muscular thickness also showed quadratic response (*P* < 0.05) (y = 164.06 + 0.6246x -0.0029x^2^; R^2^ = 0.673), with the highest value at 107.68 ppm. In the ileum, there were differences in crypt width only.

**Table 4:** Villus height, crypt depth and width, and villus: crypt ratio (mean ± standard deviation) of the intestinal mucosa of at 28 and 42 days in broilers fed diets containing different levels of protease

	Positive control	Negative control	NC + 50 ppm	NC + 100 ppm	NC + 200 ppm	CV (%)	REG
28 d							
Jejunum							
Villus height (μm)	942.04 ± 11.1^b^	965.50 ± 15.3^b^	768.29 ± 15.2^c^	1155.62 ± 15.2ª	847.76 ± 8.8^bc^	24.6	Ns
crypt depth (μm)	176.87 ± 5.3^a^	140.57 ± 4.5^bc^	148.02 ± 4.5^b^	129.99 ± 4.3^c^	150.12 ± 4.2^b^	37.9	Ns
crypt width (μm)	69.66 ± 1.3^a^	68.86 ± 1.8^ab^	64.95 ± 1.7^ab^	60.02 ± 1.2^c^	61.69 ± 1.2^b^	27.7	0.0068ª
Muscular Thickness (μm)	171.58 ± 2.9^b^	169.28 ± 3.2^b^	174.02 ± 2.6^b^	307.53 ± 5.1ª	169.52 ± 3.6^b^	32.9	< 0.0001^b^
Villus:crypt ratio	7.11 ± 0.2^c^	8.09 ± 0.3^bc^	7.85 ± 0.3^bc^	9.48 ± 0.3ª	9.10 ± 0.3^ab^	41.3	Ns
Ileum							
Villus height (μm)	626.39 ± 12.8^d^	789.63 ± 10.5^b^	889.68 ± 14.3^a^	697.55 ± 12.1^c^	757.33 ± 14.1^b^	20.9	Ns
crypt depth (μm)	83.01 ± 2.4^c^	94.60 ± 2.4^b^	110.25 ± 3.1^a^	96.67 ± 2.2^b^	96.00 ± 2.0^b^	31.3	Ns
crypt width (μm)	53.01 ± 0.9	53.76 ± 0.9	53.59 ± 0.9	52.12 ± 0.8	54.65 ± 0.9	21.3	Ns
Muscular Thickness (μm)	190.87 ± 4.1^ab^	162.09 ± 2.8^c^	171.03 ± 2.6^bc^	194.60 ± 2.7^ab^	186.17 ± 3.7^ab^	28.1	< 0.0001^c^
Villus:crypt ratio	7.12 ± 0.3^d^	8.27 ± 0.3^bc^	7.35 ± 0.2^ cd^	9.94 ± 0.3ª	9.36 ± 0.3^ab^	42.2	Ns
42 d							
Jejunum							
Villus height (μm)	1104.27 ± 14.8^b^	972.03 ± 12.1^c^	1009.19 ± 10.9^c^	1185.97 ± 13.4^a^	1112.60 ± 15.1^b^	17.7	< 0.0001^d^
crypt depth (μm)	120.68 ± 3.5^d^	145.64 ± 4.9^bc^	150.01 ± 4.6^b^	170.09 ± 5.5^a^	129.08 ± 3.4^ cd^	40.8	< 0.0001^e^
crypt width (μm)	58.17 ± 1.2^d^	74.75 ± 2.4^b^	66.47 ± 1.1^c^	96.65 ± 2.6^a^	56.14 ± 1.1^d^	31.5	Ns
Muscular Thickness (μm)	205.54 ± 5.4^b^	216.11 ± 4.7^ab^	218.65 ± 6.5^ab^	235.76 ± 5.2^a^	217.09 ± 4.2^ab^	29.6	0.0129^f^
Villus:crypt ratio	8.83 ± 0.3^a^	7.77 ± 0.3^ab^	5.96 ± 0.3^c^	8.32 ± 0.3^a^	7.20 ± 0.2^b^	46.3	Ns
Ileum							
Villus height (μm)	692.61 ± 9.3^b^	719.15 ± 10.6^b^	716.00 ± 6.2^b^	871.05 ± 9.02^a^	859.07 ± 6.50^ab^	23.95	0.0079^ g^
crypt depth (μm)	116.11 ± 4.9^a^	97.09 ± 3.4^b^	106.87 ± 3.3^ab^	99.47 ± 3.99^b^	100.91 ± 3.22^b^	36.78	Ns
crypt width (μm)	59.55 ± 1.5^a^	53.09 ± 1.4^b^	54.90 ± 1.2^b^	52.33 ± 1.19^b^	60.93 ± 1.40^a^	24.17	0.0047^ h^
Muscular Thickness (μm)	164.90 ± 3.4^d^	200.05 ± 3.5^b^	252.52 ± 3.6^a^	184.89 ± 4.19^c^	182.87 ± 4.20^c^	19.27	Ns
Villus:crypt ratio	8.07 ± 0.3^b^	9.17 ± 0.3^a^	9.03 ± 0.4^a^	8.29 ± 0.32^ab^	8.43 ± 0.30^ab^	38.02	0.034^i^

Means followed by different letters in the same line differ significantly by Tukey's test (*P* < 0.05). CV = coefficient of variation. REG = regression. Ns = not significant

^a^y = 69.258 – 0.1303x + 0.0005x^2^; R^2^ = 0.9576; optimal value = 130.3 ppm; ^b^ y = 164.06 + 0.6246x – 0.0029x^2^; R^2^ = 0.6732; optimal value = 107.68 ppm; ^c^ y = 163.35 + 0.1728x; R^2^ = 0.9684; ^d^ y = 961.57 + 1.2386x; R^2^ = 0.9904; ^e^ y = 142.14 + 0.4785x – 0.0027x^2^; R^2^ = 0.8247; optimal value = 88.61 ppm; ^f^ y = 213.48 + 0.3178x – 0.0015x^2^; R^2^ = 0.6729; optimal value = 105.93 ppm; ^g^ y = 697.42 + 1.759x – 0.0046x^2^; R^2^ = 0.7357; optimal value = 191.19 ppm; ^h^ y = 53.823- 0.0349x + 0.0003x^2^; R^2^ = 0.8558; optimal value = 58.16 ppm; ^i^ y = 9.3287 – 0.0177x + 0.000005x^2^; R^2^ = 0.7037; optimal value = 147.5 ppm

At 42 days of age, there were differences in jejunal crypt width, the V/C ratio, and the ileum crypt depth. In the jejunum, there were differences in the muscular thickness (*P* < 0.05) (y = 213.48 + 0.3178x –0.0015x^2^; R^2^ = 0.6729), with the highest value at 105.93 ppm. In the ileum, there was a linear response to protease (*P* < 0.05) (y = 163.35 + 0.1728x; R^2^ = 0.968).

At 42 days of age, villus height in the jejunum showed a linear response (*P *< 0.05) (y = 961.57 + 1.2386x; R^2^ = 0.9904), while the response of crypt depth was quadratic (*P* < 0.05) (y = 142.14 + 0.4785x –0.0027x^2^; R^2^ = 0.8247) with the highest value at 88.61 ppm protease. In the ileum, villus height displayed a quadratic response (*P* < 0.05) (y = 697.42 + 1.759x -0.0046x^2^; R^2^ = 0.7357), with the highest value at 191.19 ppm protease. Crypt width also showed a quadratic response (y = 53.823—0.0349x + 0.0003x^2^; R^2^ = 0.8558), with the lowest value at 58.16 ppm, as did the V/C ratio (y = 9.3287—0.0177x + 0.000005x^2^; R^2^ = 0.7037) with the lowest value at 147.5 ppm protease.

There were no significant differences (*P* > 0.05) in part yield of breast, thigh, wing, drumstick, but there was differences in abdominal fat pad with protease diets when evaluated in chickens at 42 days of age (Table 5).

**Table 5:** Carcass yield and cut yield (± standard error) of broilers fed diets containing different protease levels

	Positive control	Negative control	NC + 50 ppm	NC + 100 ppm	NC + 200 ppm	CV (%)	REG
Carcass	76.82 ± 0.68	78.71 ± 1.07	80.23 ± 1.06	79.33 ± 1.03	75.15 ± 0.82	4.62	Ns
Breast	38.85 ± 0.4	38.62 ± 0.68	39.89 ± 0.72	40.19 ± 0.52	39.89 ± 0.36	2.28	Ns
Thigh	15.76 ± 0.43	15.78 ± 0.38	14.91 ± 0.56	15.77 ± 0.46	15.45 ± 0.53	3.32	Ns
Wing	9.9 ± 0.15	9.92 ± 0.15	9.99 ± 0.11	9.5 ± 0.14	9.72 ± 0.15	4.48	Ns
Fat pad	2.56 ± 0.19b	2.61 ± 0.34a	2.31 ± 0.09c	2.45 ± 0.12c	2.36 ± 0.23c	1.7	Ns
Drumstick	14.42 ± 0.23	14.21 ± 0.28	14.14 ± 0.38	14.27 ± 0.34	13.89 ± 0.18	4.31	Ns

Means followed by different letters in the same line differ significantly by Tukey's test (*P* < 0.05). CV = coefficient of variation. REG = regression. Ns = not significant

There were significant differences in the meat quality in breast meat (Table 6) in the parameter b* a quadratic effect is observed in the yellow blue color.

**Table 6:** Quality parameters of meat (± standard error) of broilers fed diets containing different protease levels

	Positive control	Negative control	NC + 50 ppm	NC + 100 ppm	NC + 200 ppm	CV (%)	REG
Breast							
Ph	5.90 ± 0.02	5.93 ± 0.01	5.89 ± 0.02	5.85 ± 0.05	5.91 ± 0.02	1.58	Ns
L*	55.94 ± 0.88	58.75 ± 0.82	58.85 ± 1.00	59.55 ± 1.15	59.10 ± 0.30	4.03	Ns
a*	14.05 ± 0.54	13.49 ± 0.72	13.69 ± 0.34	13.17 ± 0.76	13.59 ± 0.51	11.38	Ns
b*	15.84 ± 1.29	19.21 ± 0.74	16.89 ± 0.63	16.98 ± 1.00	15.80 ± 0.45	13.8	0.012^a^
CWL%	20.55 ± 1.04	25.50 ± 2.65	26.92 ± 3.43	24.86 ± 2.59	22.78 ± 2.27	28.02	Ns

L*, lightness; a*, redness; b*, yellowness; CWL, cooking weight loss; Means followed by different letters in the same line differ significantly by Tukey's test (*P*<0.05). CV = coefficient of variation. REG = regression. Ns = not significant

## Discussion

The increasing linear response to consumption from day 12 to 21 was due to the high concentrations of protease in the diet, which increased the availability of amino acids that promote consumption (Yuan et al., 2017). Ciftci and Ceylan (2004) reported that threonine included at 0.78% in broiler diets increased feed intake by 18% in the initiation phase; likewise, Corzo et al. (2005) reported increases in feed intake up to 18% in the growth and finishing phases with the inclusion of 0.72% threonine in the diet, while the inclusion of proline, alanine, and aspartic acid increased feed intake by 9%. The inclusion of protease in the diet improves weight gain in animals, which positively influences feed conversion (Dessimoni et al., 2019). This is evidenced in the period from 12 to 21 days. The same effect occurs for weight gain and feed consumption in the period from 29 to 42 d, so we can agree with Angel et al. (2011) that the addition of protease to the broiler diet improves production parameters.

The behavior observed in MS at 28 days is that protease favors the breakdown of carbohydrate-protein bonds and thus improves the availability of absorbable carbohydrates in the small intestine (Jabbar et al., 2021). The increase in protein digestibility at 28 days represents 14% more protein when compared to the positive control. Cho et al. (2020) reported that protease utilization increases protein digestibility or the protein digestibility coefficient. In addition, Carvalho et al., (2020) reported increased expression of genes that activate protein transport channels in enterocytes whenever protease is added to the diet. Thus, the addition of protease to the diet increases the availability of the absorbable forms of proteins by enterocytes (tripeptides, dipeptides, and amino acids) in the lumen of the small intestine (Peluzio & Batista, 2008).

Differences in crypt size (P < 0.05) in the jejunum at 28 days indicated slower cell turnover (Khodambashi et al., 2012) and a lower nutrient demand for crypt cell growth (Khodambashi et al., 2012). Furthermore, it is presumed that a higher availability of essential amino acids such as lysine, methionine, and valine affected the muscular layer of the jejunum and ileum at 28 days of age, in agreement with reports by Kamel et al. (2015), where high levels lysine and methionine in chickens induced a significant increase in intestinal size.

The effect on villus size in the jejunum and ileum at 42 days was probably due to the fact that the inclusion of proteases increases the amount of digestible peptides available for villus growth (Santos et al., 2018). These results suggest that the use of protease significantly influences the size and integrity of intestinal villi, also influencing crypt size and the muscular V/C ratio. In broilers, larger villi increase the absorption capacity in the small intestine (Khodambashi et al., 2012), and are related to higher V/C ratios, indicating good intestinal health and better production parameters Furthermore, this can be explained by the change in the protein degradation sites, where this will occur rapidly in more anterior segments of the intestine (Duodenum, jejunum and ileum), which will decrease the excess of fermentable substrate by the microbiota. of posterior segments, producing fermentable oligosaccharides only from inert fibrous plant material, which benefits intestinal pH and the appearance of beneficial microorganisms and the good development of enterocytes (Bedford, 2000). Zuo et al. (2015) reported that diets including protease increased the V/C ratio index; they further indicated that proteases are associated with increased growth and intestinal efficiency, for example, by reducing inflammation (Kamel et al., 2015).This indicating that with the inclusion of exogenous protease, excess protein digested in the intestine can be stabilized as well as regulating pathogenic microorganisms and thus promoting the growth of beneficial microorganisms, which inhibit the proliferation and development of pathogenic agents. (Cowieson & Kluenter, 2018).

The effect on fat pad in the diets with protease at 42 days was probably due to an increase in the activity of carnitine, which is a quaternary ammonium amino acid derived from hepatic, renal, and cerebral metabolism from dietary amino acids such as methionine and lysine (Cave et al., 2008); which could be increased and thus stimulate the synthesis of carnitine. Likewise, the quadratic behavior observed in the parameter b* in the breast can be explained at 42 days, where in the CN and CN + 200 diets the yellow color of the meat decreased due to a catabolism of the cartenoid lipid components.

## Conclusion

The use of proteases in broiler diets improves parameters such as weight gain, feed conversion, histomorphometry parameters, meat quality, and carcass yield. This shows that the inclusion of proteases is an opportunity to decrease the amount of protein in the diet without affecting the animal's development. This will reduce production costs and results in a profit opportunity for broiler producers.

Furthermore, it is pertinent for future research to delve into the relationship between proteases with the color of the meat and meat quality. In addition, the interaction of pancreatic enzymes such as lipase and colipase, amino acid and lipid digestibility can be evaluated in order to make a deeper connection of the results.

## Data Availability

Not applicable.
